# Single-cell transcriptome profiling reveals dynamic cell populations and immune infiltration in cerebral cavernous malformation

**DOI:** 10.3389/fimmu.2025.1592343

**Published:** 2025-05-30

**Authors:** Zhiguang Han, Chengxu Lei, Zhenyu Zhou, Yutong Liu, Yuanli Zhao, Shihao He

**Affiliations:** ^1^ Department of Neurosurgery, The First Hospital of Qinhuangdao, Hebei, China; ^2^ Department of Neurosurgery, Peking Union Medical College Hospital, Peking Union Medical College and Chinese Academy of Medical Sciences, Beijing, China; ^3^ Department of Neurosurgery, Beijing Tiantan Hospital, Capital Medical University, Beijing, China

**Keywords:** cerebral cavernous malformation, single-cell RNA sequencing, multiplex fluorescent immunohistochemistry, cell populations, immune infiltration

## Abstract

**Introduction:**

The cellular subpopulations and signaling pathways in the pathological tissues of cerebral cavernous malformation (CCM) remain incompletely understood. To gain a deeper understanding of the pathogenesis of CCM, we aimed to comprehensively map the cellular subpopulations and signaling pathway alterations in the pathological tissues of sporadic CCM patients.

**Methods:**

Lesional brain vascular tissues from CCM patients and normal brain vascular tissues from controls were collected. Multiplex fluorescent immunohistochemistry and single-cell RNA sequencing were performed on the lesional tissues. Differential gene expression, pathway enrichment analysis, and cell-cell communication analysis were conducted to investigate disease-related changes.

**Results:**

We identified 8 major cell types in the lesion tissues of CCM patients. We observed an increased proportion of monocytes, neutrophils, and NK cells in the lesion tissues of CCM patients. Twenty-eight significantly differentially expressed genes were identified, and pathways such as NK cell-mediated cytotoxicity showed alterations. Cell-cell communication analysis revealed an increase in both the types and strength of communication between cells in the CCM lesion tissues.

**Conclusion:**

This study provides the single-cell transcriptomic analysis of CCM lesions, revealing increased monocytes, neutrophils, and NK cells, along with dysregulated gene expression and signaling pathways. Enhanced intercellular communication, particularly via VEGF and ADGRE5 pathways, highlights potential therapeutic targets for CCM.

## Introduction

Cerebral cavernous malformation (CCM) is a common type of cerebral vascular malformation, characterized by its distinctive cavernous structure and associated potential for fatal risks (1). The pathological features of CCM typically include vascular dilation, reduced endothelial cell-cell contact, and disruption of the blood-brain barrier (2). These characteristics make CCM prone to rupture, leading to intracerebral hemorrhage and other neurological damage (3). Currently, the pathogenesis of CCM remains unclear, and there is no targeted pharmacological therapy. The existing treatment primarily focuses on surgical resection but the high risk associated with deep lesions underscores the urgent need for the development of targeted therapeutic strategies (4).

CCM can be categorized into familial and sporadic forms. Familial CCM has been associated with mutations in the CCM1 (5), CCM2, and CCM3 genes, while sporadic CCM is linked to mutations in the MAP3K3 and PIK3CA genes (6–9). Recent research has highlighted the important role of the ERK-MAPK and PI3K signaling pathways in the pathogenesis of CCMs. For instance, studies have shown that aberrations in the ERK-MAPK pathway can influence cellular proliferation and survival in CCM endothelial cells (10, 11).It was discovered that the development of CCM is related to inflammation, with CCM3 gene potentially inducing an inflammatory response and immune cell infiltration by upregulating inflammation-related genes in endothelial cells of mice (12). Previous study identifies novel regulatory signaling networks and key cellular factors associated with CCM signaling complex (13). Koskimäki et al., through analysis of circulating miRNAs, identified mmu-miR-3472a as a potential regulator of the Cand2 gene, influencing the inflammatory process in CCM development (14). However, current studies are limited to animal CCM tissues or human peripheral blood samples, with insufficient in-depth exploration of human CCM samples.

Advancements in single-cell RNA sequencing (scRNA-seq) technology have provided unprecedented resolution for systematically analyzing cell subpopulations and reconstructing cell-cell interaction networks in complex tissues (15). RNA sequencing studies on patient samples can help identify mutated genes related to CCM but do not offer insights into the specific expression changes of these genes within tissue subpopulations (16). ScRNA-seq can accurately identify functionally abnormal cell populations in the disease microenvironment and reveal the gene regulatory dynamics driving phenotypic changes (17). ScRNA-seq has already been applied to other vascular diseases. Jennifer et al. utilized scRNA-seq to identify 13 immune cell subpopulations in atherosclerotic tissue (18). Zhang et al. discovered that blocking the ALK5-SMAD2 signaling pathway effectively prevents arteriovenous malformations (AVM) (19). Johnathan et al. analyzed multiple CCM-deficient animal models in 9 independent studies by using comparative genomics methods (20). However, though transcriptome analysis of CCM has been reported, single-cell level studies in the field of CCM are yet to be conducted (21).

The objective of this study is not only to explore the cellular composition and transcriptomic characteristics of CCM through immunofluorescence staining and single-cell sequencing technology but also to provide new insights for understanding the molecular mechanisms of CCM and identifying potential therapeutic targets. By revealing the cellular heterogeneity of CCM, we aim to provide a theoretical basis for the early diagnosis and development of treatment strategies for CCM.This study is the first to directly analyze the cellular heterogeneity of human cerebral cavernous malformation (CCM) lesions using single-cell transcriptomic sequencing and multiplex immunofluorescence staining, thus avoiding biases from animal models. Our analysis involved 1 sporadic CCM sample and 3 STA samples from healthy controls, and revealed a dynamic network of interactions among endothelial, immune, and stromal cells within the CCM microenvironment, addressing the limitations of traditional RNA sequencing in examining cell-specific expression. We identified eight cell subpopulations, with an increased proportion of monocytes, neutrophils, and NK cells in the lesions. Additionally, we discovered 28 significant genes and performed GO and KEGG pathway enrichment analyses for each subpopulation. Cell-cell communication analysis demonstrated an increase in both the types and strength of communications in CCM lesions, providing new insights into the pathogenesis and treatment development of the disease.

## Materials and methods

### Tissue sample collection and processing

CCM tissue samples and clinical data were obtained with approval from the Ethics Committee of Peking Union Medical College Hospital (approval number I-24PJ2435). 3 STA tissue samples were obtained from epilepsy patients during surgery. All tissue samples were collected from patients undergoing neurosurgical procedures, who had signed written informed consent prior to surgery, permitting the use of excised tissue for research purposes. Preoperative diagnosis of cerebral CCM was confirmed through angiography. During surgery, malformed vascular segments were precisely resected under a surgical microscope, avoiding hemorrhagic and necrotic regions. Each sample was approximately 5–10 mm³ to ensure uniformity. The excised vascular tissue was immediately washed three times in Dulbecco’s Phosphate-Buffered Saline (DPBS) to remove blood and debris, and then transported on ice for processing within 2 hours. If immediate processing was not possible, samples were stored in 4°C DPBS (no longer than 6 hours).

Under a sterile laminar flow hood, the tissue was cut into approximately 0.5 mm³ pieces and incubated in enzyme digestion solution (collagenase II 1 mg/ml, DNase 50 µg/ml, hyaluronidase 0.1 mg/ml) at 37°C for 30–60 minutes, with gentle pipetting every 10 minutes to promote dissociation. The digested suspension was filtered through a 70 µm mesh filter to remove undigested fragments and collected into 15 ml centrifuge tubes. Red blood cell contamination was removed using red blood cell lysis buffer, and cell viability was assessed using trypan blue staining. Live cells (trypan blue-negative) were sorted using flow cytometry (FACS) to ensure high-quality sequencing. Cell concentration was determined using a hemocytometer to ensure each sample contained 10^5^-^106^ cells/ml.

### Multiplex fluorescent immunohistochemistry

In this study, we employed conventional paraffin sectioning for tissue processing. The specific steps were as follows: First, the paraffin sections were dewaxed and washed in distilled water for 5 minutes. The sections were then placed in a retrieval solution, ensuring complete coverage of the tissue, and treated under high pressure at 240°C for 4 minutes, until the water boiled. The sections were subsequently washed with PBS buffer three times, 5 minutes each. Next, 3% hydrogen peroxide was added, and the sections were incubated at room temperature for 5 minutes. After additional PBS washing, the sections were incubated with blocking solution at 37°C for 30 minutes. The primary antibody working solution (CD31, ab9498, CY3, dilution 1:50, chromogen concentration 1:200) was added and incubated overnight at 4°C. On the following day, the sections were washed with PBS and incubated with secondary antibody (anti-mouse/rabbit IgG polymer, at 37°C for 30 minutes). After further PBS washing, 488 chromogen was added and incubated for 3 minutes at room temperature. The procedure was repeated for each antibody labeling in the following order: primary antibody CD31 (ab9498, CY3, 1:50, 1:200), primary antibody α-SMA (ab124964, 488, 1:5000, 1:50), primary antibody Claudin5 (ab131259, 594, 1:1000, 1:100), primary antibody ZO-1 (33-9100, 700, 1:25, 1:50), primary antibody Vimentin (CY5, 1:1000, 1:200). After antibody labeling, sections were washed again in PBS and mounted with DAPI for nuclear staining.

### Reference genome and transcript annotation file download and processing

The human reference genome sequence (GRCh38 version) was obtained from the Ensembl database (22) (https://useast.ensembl.org/index.html) and downloaded in FASTA format (Homo_sapiens.GRCh38.dna.primary_assembly.fa). The corresponding gene annotation file (Homo_sapiens.GRCh38.112.gtf) was also obtained. The annotation file was filtered using the CellRanger tool (23) (version 8.0.1, https://www.10xgenomics.com/support/software/cell-ranger/latest) mkgtf module, retaining only protein-coding gene exons, followed by genome index construction using the mkref module.

### Single-cell upstream analysis

The raw data (FASTQ format) from the 5’ single-cell RNA sequencing of samples STA1, STA2, STA4, and CCM1 were processed using the CellRanger count pipeline for sequence alignment, quality filtering, barcode identification, and UMI quantification.

### Single-cell data processing and cell annotation

Quality control was performed using the Seurat package (24) (version 5.1.0, https://github.com/satijalab/seurat). The filtering criteria were 50 < nFeature_RNA < 7000 and percent.mt < 5%. Principal component analysis (PCA) was conducted, followed by batch effect correction using the Harmony algorithm. Cell clustering was performed with a resolution parameter of 0.6, and cell type annotation was carried out using the SingleR tool (25) (version 2.4.1, https://www.bioconductor.org/packages/release/bioc/html/SingleR.html).

### Differential analysis

Differentially expressed genes in the CCM group compared to the control group were identified using the FindMarkers algorithm. The log2 fold change (log2FC) and p-value were extracted. The top 5 differentially expressed genes with the highest significance in each cell type were selected for integration and visualized using DotPlot, illustrating their expression patterns across different sample types and cell subpopulations.

### GO and KEGG enrichment analysis

ClusterProfiler (26) (version 4.10.0, https://bioconductor.org/packages/release/bioc/html/clusterProfiler.html) was used to perform two types of analysis: (1) KEGG pathway gene set enrichment analysis (GSEA) for all differentially expressed genes (27); (2) GO functional annotation of genes with |log2FC| > 2 and p-value < 0.05 (28).

### Cell communication analysis

CellChat (29) (version 1.5.0, https://github.com/sqjin/CellChat) was used to construct a cell communication network and quantitatively compare the characteristics of cell interactions and the differential activity of signaling pathways between different samples, providing a comprehensive understanding of the impact of disease status on intercellular communication patterns.

## Results

### Multiplex fluorescent immunohistochemistry

**Figure 1:**
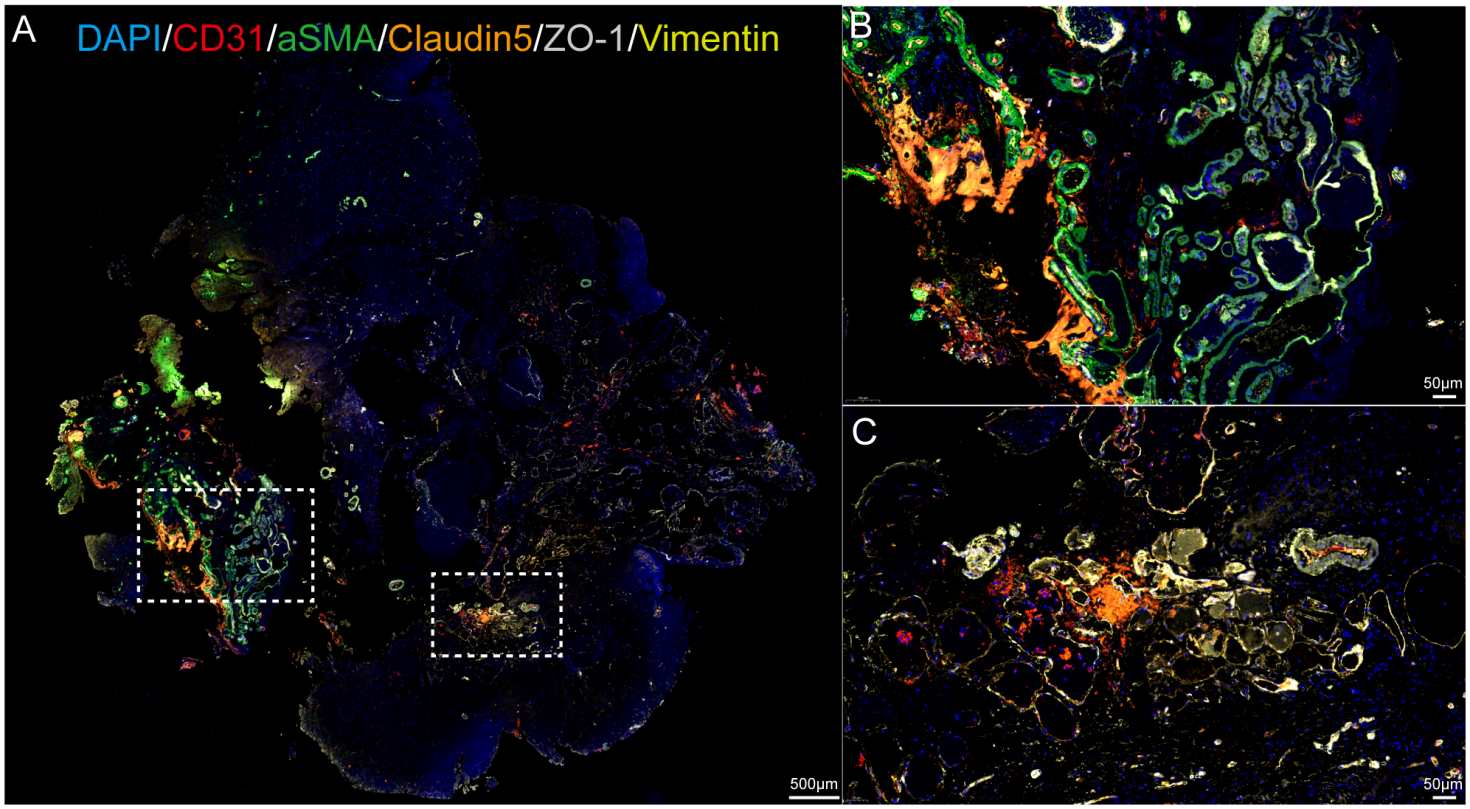
Multiplex fluorescent immunohistochemistry result. CD31 labels endothelial cells, α-SMA labels vascular smooth muscle cells, DAPI labels DNA, Claudin5 and ZO-1 label the blood-brain barrier, and Vimentin labels fibroblasts. Scale bar=500 and 50 μm.

We performed fluorescent immunostaining and found that the expression of tight junction proteins Claudin5 and ZO-1 in endothelial cells was downregulated within the CCM lesions, as well as the downregulation of Vimentin expression in fibroblasts (**Figure 1**). Specifically, the number of endothelial cells, smooth muscle cells and fibroblasts was decreased in CCM sample compared to control samples (**Supplementary Figure S6**).

### Single-cell data processing and cell annotation

All the processed and raw data of single-cell sequencing for 3 STA and 1 CCM samples were uploaded to GEO database as GSE294555. After quality control, a total of 40,010 cells were included for subsequent analysis (**Supplementary Figure S1A**), which consisted of 11,045 cells from CCM1, 5,451 cells from STA1, 11,130 cells from STA2, and 12,384 cells from STA4. PCA was performed using the top 3,000 highly variable genes, and we observed that the variance in the principal components plateaued when the number of components reached 20 (**Supplementary Figure S1B**). Based on this, we used 20 principal components for Harmony batch effect correction and visualized the sample mixing with UMAP plots before and after batch effect correction, as shown in **Supplementary Figures S1C, D**.

**Figure 2:**
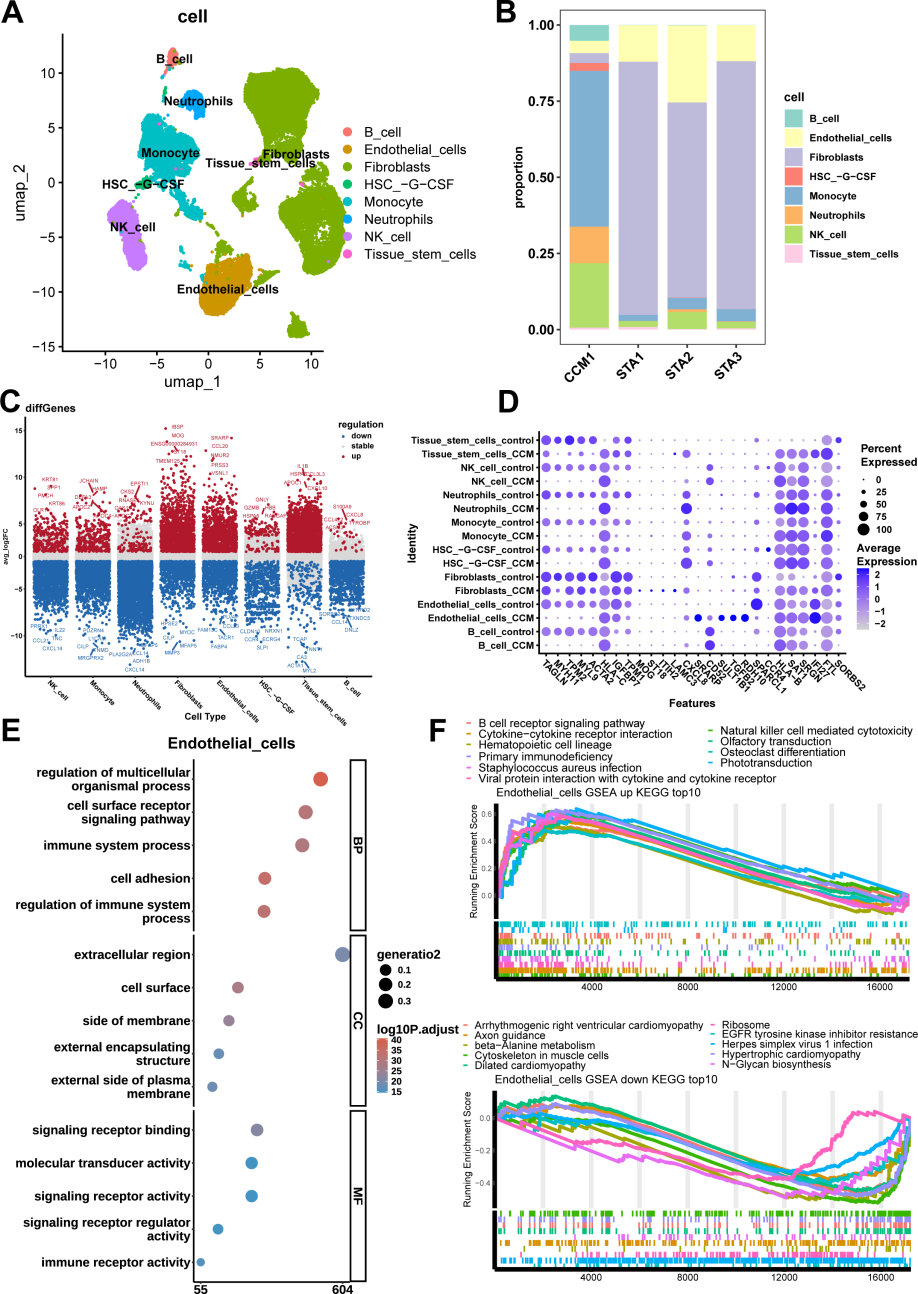
Cell types, distribution, significant differential genes, GO and KEGG enrichment results of Endothelial cells. **(A)** UMAP plot annotated by cell type shows 8 cell types, including NK cells, Monocytes, Neutrophils, Fibroblasts, Endothelial cells, HSC-G-CSF (hematopoietic stem cells mobilized to peripheral blood by granulocyte colony-stimulating factor), tissue stem cells, and B cells. **(B)** cell type proportion stacked bar plot. CCM sample shows more Monocytes, Neutrophils, NK cells and less Fibroblasts, Endothelial cells. **(C)** Log2FC scatter plot for differential genes in each cell type, with red representing upregulated genes, blue representing downregulated genes, and gray indicating no significant difference. **(D)** Bubble plot of the 28 most significant differential genes, with genes on the x-axis and cell type sample group on the y-axis. **(E)** GO enrichment analysis of differential genes in Endothelial cells. The most significant signaling pathways for the BP, CC, and MF terms are regulation of multicellular organismal processes, cell surface and signaling receptor binding. **(F)** Enrichment plot of the top 10 most significant upregulated and downregulated pathways in Endothelial cells. The most significant upregulated and downregulated signaling pathway are Natural Killer cell-mediated cytotoxicity and EGFR tyrosine kinase inhibitor resistance.

Subsequently, cell clustering and annotation were performed using the 20 Harmony-corrected coordinates (**Figure 2A**). The annotation results revealed 8 distinct cell types: NK cells, monocytes, neutrophils, fibroblasts, endothelial cells, HSC-G-CSF (hematopoietic stem cells mobilized to peripheral blood by granulocyte colony-stimulating factor), tissue stem cells, and B cells. We observed that the distribution of cell types varied between samples, with the three STA samples having a similar cell composition, with the most abundant cell type being fibroblasts. In contrast, the CCM sample was predominantly composed of Monocytes (**Figure 2B**). Additionally, the proportion of immune cells was significantly higher in CCM samples compared to STA samples (**Figure 2B**).

### Differential analysis

Differential analysis was performed using a threshold of |log2FC| > 2 and p-value < 0.05, and the log2FC scatter plot for each cell type’s differential genes was obtained, marking the top 5 most significantly upregulated and downregulated genes (**Figure 2C**). The results showed that NK cells had 290 upregulated and 1,633 downregulated genes, monocytes had 293 upregulated and 1,614 downregulated genes, neutrophils had 146 upregulated and 3,540 downregulated genes, fibroblasts had 1,249 upregulated and 832 downregulated genes, endothelial cells had 1,076 upregulated and 925 downregulated genes, HSC-G-CSF had 142 upregulated and 631 downregulated genes, tissue stem cells had 1,721 upregulated and 201 downregulated genes, and B cells had 32 upregulated and 1,025 downregulated genes. As there were overlapping genes across different cell types, the 28 most significant genes across all cell types were identified: TAGLN, MYH11, TPM2, MYL9, ACTA2, HLA-C, IGFBP7, TPM1, MOG, ST18, ITIH2, LAMC3, CXCL8, SRARP, CD52, SULT1B1, TGFB2, RDH10, SPARCL1, CCR4, HLA-B, SAT1, SRGN, IFI27, FTL, SORBS2 (**Figure 2D**). Among them, CXCL8, also known as IL-8, is a chemokine that plays a critical role in inflammatory responses, and angiogenesis (30, 31).

### GO and KEGG enrichment analysis

KEGG gene set enrichment analysis (GSEA) was performed with a threshold of |NES| > 1 and adjust.p-value < 0.05. The most significantly enriched pathways were identified for each cell type.

For endothelial cells in the CCM group compared to the STA group, 64 significantly enriched pathways were identified, including 37 upregulated pathways (with the most significant being NK cell-mediated cytotoxicity) and 27 downregulated pathways (with the most significant being EGFR tyrosine kinase inhibitor resistance). **Figure 2F** shows the top 10 most significant upregulated and downregulated pathways.

**Figure 3:**
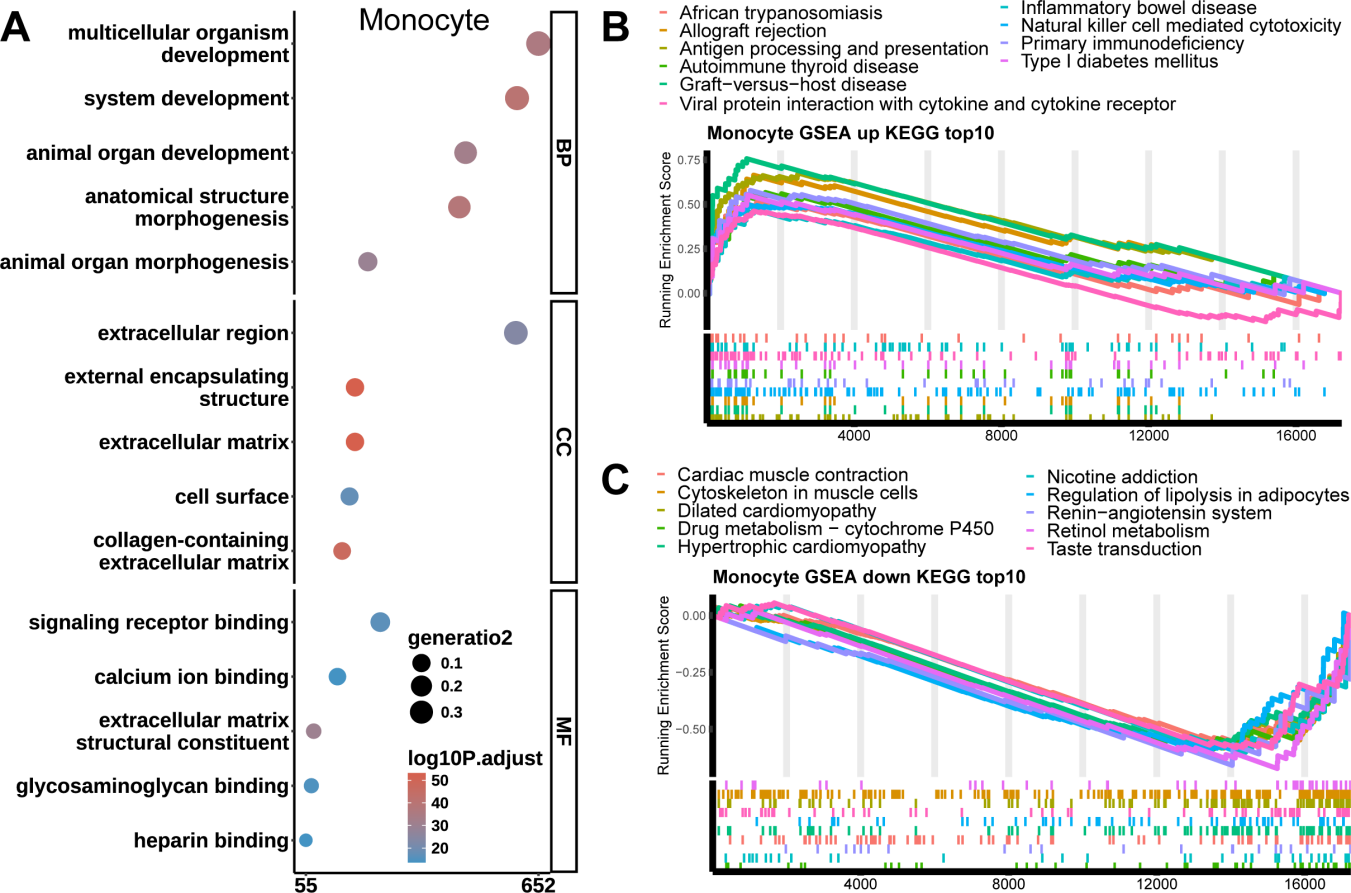
GO and KEGG enrichment results of Monocytes. **(A)** GO enrichment analysis results for differential genes in Monocytes. The most significant signaling pathways for the BP, CC, and MF terms are system development, extracellular matrix, and extracellular matrix structural constituent. **(B)** Top 10 upregulated pathways in Monocytes. The most significant upregulated signaling pathway is Antigen processing and presentation. **(C)** Top 10 downregulated pathways in Monocytes. The most significant downregulated signaling pathway is Drug metabolism - cytochrome P450.

For monocytes in the CCM group compared to STA, 67 significantly enriched pathways were found, with 24 upregulated (most significantly antigen processing and presentation) and 43 downregulated (most significantly drug metabolism - cytochrome P450). **Figures 3B, C** show the top 10 most significant upregulated and downregulated pathways.

For fibroblasts in the CCM group, 79 significantly enriched pathways were identified, including 60 upregulated (most significantly NK cell-mediated cytotoxicity) and 19 downregulated (most significantly Axon guidance). **Supplementary Figures S3A, B** show the top 10 upregulated and downregulated pathways.

For neutrophils in the CCM group, 68 significantly enriched pathways were identified, with 11 upregulated (most significantly Graft-versus-host disease) and 57 downregulated (most significantly Taste transduction). **Supplementary Figures S4A, B** show the top 10 upregulated and downregulated pathways.

For NK cells, 108 significantly enriched pathways were found, with 21 upregulated (most significantly antigen processing and presentation) and 87 downregulated (most significantly arrhythmogenic right ventricular cardiomyopathy). **Supplementary Figures S5A, B** show the top 10 upregulated and downregulated pathways.

We selected differential genes with |log2FC| > 2 and p-value < 0.05, followed by GO enrichment analysis. The significant GO terms were further filtered based on the criteria of p.adjust < 0.05 and count >= 2.

For endothelial cells in the CCM group compared to the STA group, a total of 1,918 significantly enriched GO terms were identified, including 1,652 Biological Process (BP) terms (the most significant being regulation of multicellular organismal processes), 165 Cellular Component (CC) terms (the most significant being cell surface), and 101 Molecular Function (MF) terms (the most significant being signaling receptor binding). **Figure 2E** shows the top 5 most significant enriched terms in each category.

For monocytes in the CCM group compared to the STA group, 1,373 significantly enriched GO terms were identified, including 1,128 BP terms (the most significant being system development), 137 CC terms (the most significant being extracellular matrix), and 108 MF terms (the most significant being extracellular matrix structural constituent). **Figure 3A** shows the top 5 most significant enriched terms in each category.

In the CCM group, compared to the STA group, B cells had 279 significantly enriched GO terms, including 214 BP terms (the most significant being anatomical structure morphogenesis), 50 CC terms (the most significant being collagen-containing extracellular matrix), and 15 MF terms (the most significant being extracellular matrix structural constituent). **Supplementary Figure S2A** shows the top 5 most significant enriched terms in each category.

For fibroblasts in the CCM group compared to the STA group, 1,666 significantly enriched GO terms were identified, including 1,396 BP terms (the most significant being cell adhesion), 143 CC terms (the most significant being cell surface), and 127 MF terms (the most significant being signaling receptor binding). **Supplementary Figure S2B** shows the top 5 most significant enriched terms in each category.

For HSC-G-CSF in the CCM group compared to the STA group, 174 significantly enriched GO terms were found, including 94 BP terms (the most significant being actin filament-based processes), 65 CC terms (the most significant being actin cytoskeleton), and 15 MF terms (the most significant being extracellular matrix structural constituent). **Supplementary Figure S2C** shows the top 5 most significant enriched terms in each category.

For neutrophils in the CCM group compared to the STA group, 984 significantly enriched GO terms were identified, including 751 BP terms (the most significant being multicellular organism development), 160 CC terms (the most significant being collagen-containing extracellular matrix), and 73 MF terms (the most significant being extracellular matrix structural constituent). **Supplementary Figure S2D** shows the top 5 most significant enriched terms in each category.

For NK cells in the CCM group compared to the STA group, 1,663 significantly enriched GO terms were identified, including 1,426 BP terms (the most significant being anatomical structure morphogenesis), 154 CC terms (the most significant being collagen-containing extracellular matrix), and 83 MF terms (the most significant being extracellular matrix structural constituent). **Supplementary Figure S2E** shows the top 5 most significant enriched terms in each category.

For tissue stem cells in the CCM group compared to the STA group, 1,550 significantly enriched GO terms were identified, including 1,285 BP terms (the most significant being regulation of immune system processes), 167 CC terms (the most significant being vesicle), and 98 MF terms (the most significant being protein-containing complex binding). **Supplementary Figure S2F** shows the top 5 most significant enriched terms in each category.

### Cell communication analysis

**Figure 4:**
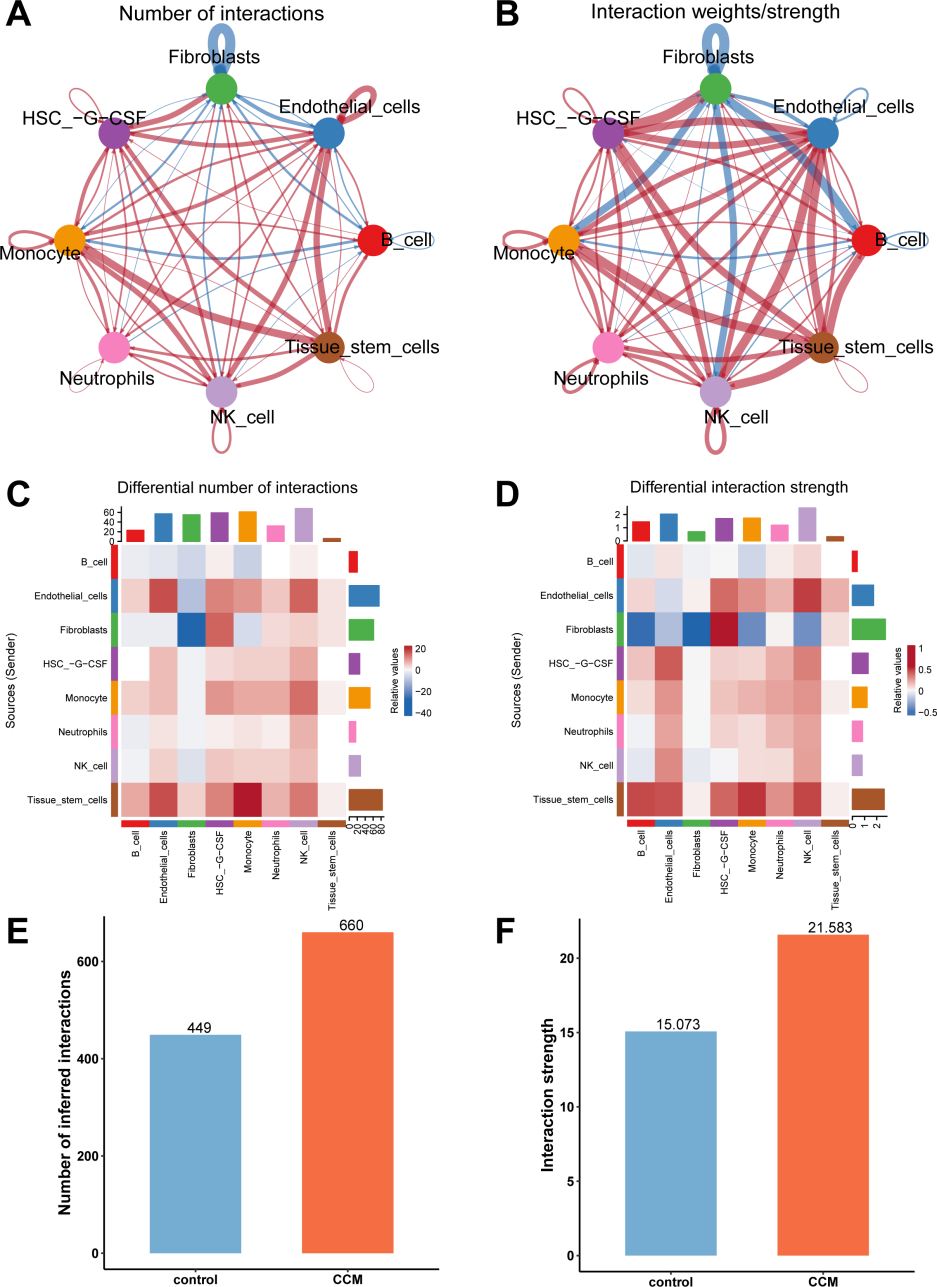
Cell communication analysis results. **(A)** Network plot comparing cell communication types between CCM and control samples, with red indicating more types in CCM and blue indicating fewer types. **(B)** Network plot comparing communication intensity between CCM and control samples, with red indicating stronger communication in CCM and blue indicating weaker communication. **(C)** Heatmap comparing the number of cell communication types in CCM vs. control. **(D)** Heatmap comparing communication strength in CCM vs. control. **(E)** Comparison of the number of cell communication types. CCM samples had 660 cell communication types while control had 449. **(F)** Comparison of the total communication strength. CCM samples had an interaction strength of 21.583 while control had 15.073.

Finally, we conducted a cell communication analysis and compared the overall cell communication patterns between CCM and control samples (**Figures 4A, B**). The results showed that in CCM samples, cell communication related to Fibroblasts was reduced, while communication between most other cell types was increased in both quantity and strength (**Figures 4C, D**). When comparing all cell types across different sample types, CCM samples showed increased types and intensity of cell communications (**Figures 4E, F**). Specifically, CCM samples had 660 cell communication types, with a total communication strength of 21.583, compared to 449 types and a strength of 15.073 in control samples.

**Figure 5:**
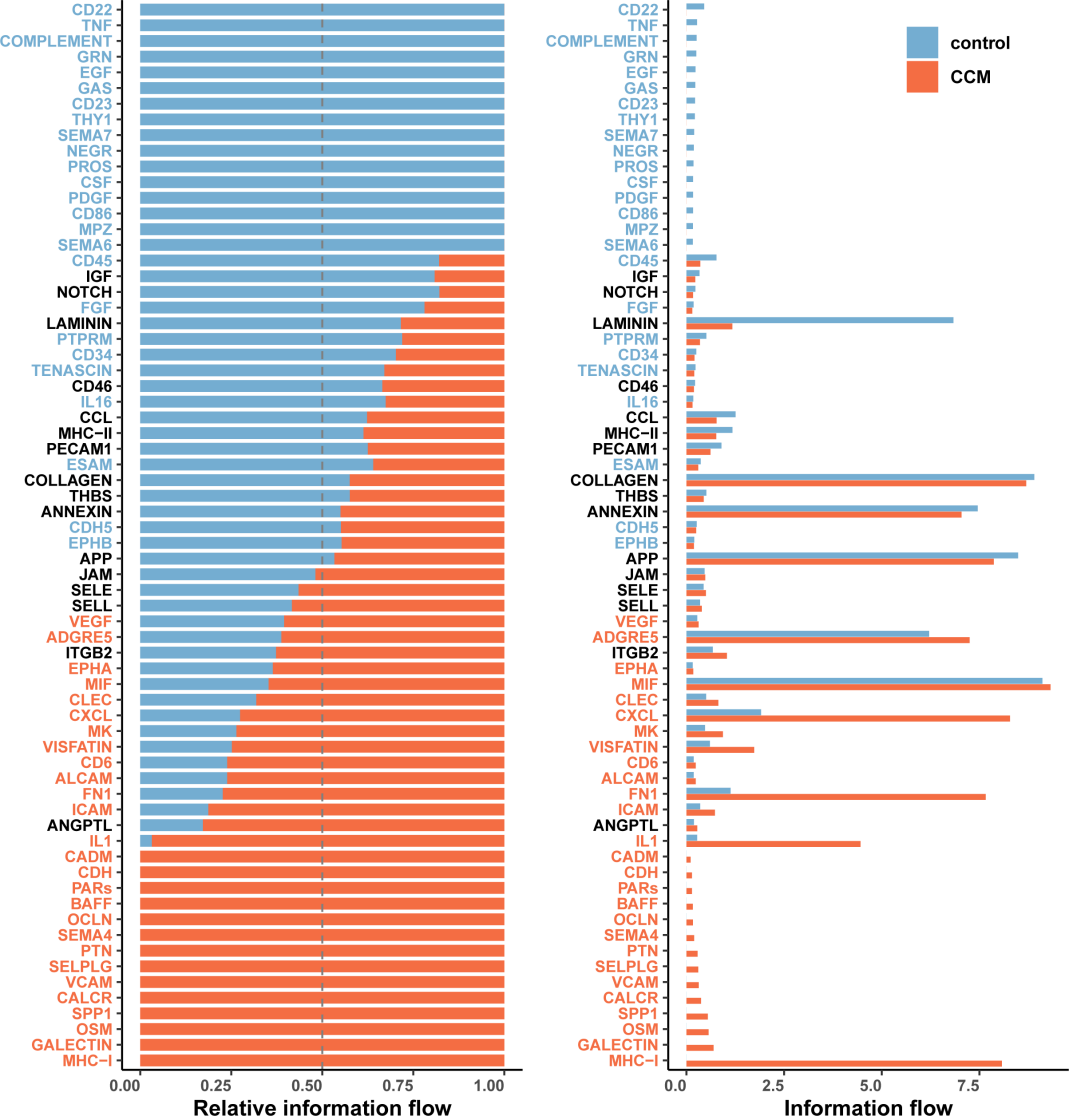
Significant communication pathways between CCM and control samples. Left plot shows proportion of communication pathway strength. Right plot shows communication pathway strength values. Orange indicates significantly activated pathways in CCM relative to controls while blue indicates significantly suppressed pathways. Significantly activated pathways include VEGF, ADGRE5, EPHA, MIF, CLEC, CXCL, MK, VISFATIN, CD6, ALCAM, FN1, ICAM, IL1, CADM, CDH, PARs, BAFF, OCLN, SEMA4, PTN, SELPLG, VCAM, CALCR, SPP1, OSM, GALECTIN, and MHC-I. Significantly suppressed pathways include CD22, TNF, COMPLEMENT, GRN, EGF, GAS, CD23, THY1, SEMA7, NEGR, PROS, CSF, PDGF, CD86, MPZ, SEMA6, CD45, FGF, PTPRM, CD34, TENASCIN, IL16, ESAM, CDH5, EPHB.

Next, we further analyzed the differences in communication pathways between CCM and control samples at the pathway level (**Figure 5**). The left panel shows the proportion of communication pathway strength, while the right panel shows the pathway strength values. The results revealed that the following communication pathways were significantly activated VEGF, ADGRE5, EPHA. The following pathways were significantly suppressed CD22, TNF, COMPLEMENT.

## Discussion

Cerebral cavernous malformation is a high-risk cerebrovascular disease, and previous research has not deeply explored the cellular subpopulations and signaling pathways within patient lesion tissues. This study is the first to directly analyze the cellular heterogeneity of human CCM lesions obtained through surgery, combining single-cell transcriptomic sequencing and multiplex immunofluorescence staining techniques. We identified 8 cell subpopulations and found an increased proportion of monocytes, neutrophils, and NK cells in the lesion tissues of CCM patients. We then identified 28 most significant genes and analyzed the GO and KEGG pathway enrichment results for each cell subpopulation. Finally, we performed a cell-cell communication analysis, which revealed that both the number of cell communication types and communication strength were increased in the CCM lesion tissue. These results provide new insights for future research into the pathogenesis of CCM.

CXCL8 promotes neutrophil chemotaxis, endothelial cell proliferation, and changes in vascular permeability by binding to receptors CXCR1/CXCR2, activating downstream signaling pathways such as the MAPK and Rho-GTPase pathways (32). Li et al. found that CXCL8 can promote endothelial cell proliferation and regulate angiogenesis (11). There is currently no direct research on CXCL8 and cerebral cavernous malformation (CCM), exploring the pathways involving CXCL8 reveals some associations. Ma et al. found a link between the ERK-MAPK cascade and CCM, regulated by PDCD10 through MST4 (10). Huo et al. discovered that MAPK-mutant mice develop CCM-like lesions, and activation of PI3K can sustain these lesions (33). Additionally, Knall et al. found that CXCL8 can activate the MAPK pathway through PI3K (34). On the other hand, Whitehead et al. found that CCM2 deficiency leads to activation of Rho-A GTPase, and inhibiting Rho-A GTPase rescued the CCM2-deficient mouse phenotype (35). Schraufstatter et al. also found that CXCL8 promotes Rho activation (36). These findings are consistent with our observation that CXCL8 expression is upregulated in all 8 cell subpopulations, suggesting that upregulation of CXCL8 may activate the PI3K/MAPK and Rho-GTPase pathways, both of which contribute to the progression of CCM.

Endothelial dysfunction or immune cell dysfunction may be related to the mechanism of CCM lesions (37, 38). Scimone et al. analyzed endothelial cells isolated from CCM lesions and found that the genes were enriched in pathways related to angiogenesis and other processes (39). Regarding endothelial issues, evidence supports two opposing ideas: one hypothesis states that loss of endothelial cells via disrupted beta1 integrin signaling causes CCM lesions (40–42), while the other view is that lesion formation results from an overgrowth of endothelial cells, also linked to beta1 integrin disruption (43–46). At present, the endothelial cell models derived from iPSCs related to CCM have dysregulation of signaling pathways involved in the pathogenesis (47). This study showed that endothelial cells decreased in CCM relative to healthy controls, which support the first hypothesis. For immune-related hypotheses, we found increase of different kinds of immune cells in CCM tissue than healthy controls and upregulation of antibody synthesis and secretion, which suggest inflammation in CCM tissue This could be an evidence that support the involvement of inflammation in CCM progression.

We found that the proportion of monocytes, NK cells, and neutrophils in CCM lesion tissues was significantly increased. Monocytes exhibited upregulation of genes such as JCHAIN and downregulation of genes such as MRGPRX2. JCHAIN encodes the J chain, a key molecule for the polymerization of secretory IgA and IgM, which play important roles in mucosal immunity and systemic inflammatory responses (48). Upregulation of JCHAIN expression may lead to abnormal immunoglobulin production in monocytes, influencing the local inflammatory microenvironment in CCM lesions. TNMD is a glycoprotein mainly expressed in tendons and cartilage, involved in extracellular matrix (ECM) remodeling and regulation of angiogenesis (49). Its downregulation may affect the stability of ECM in monocytes and, in turn, impact immune deposition in CCM lesion tissues. Monocytes also exhibited upregulation of KRT81 and downregulation of CXCL14. KRT81 (Keratin 81), a member of the type II keratin family, is involved in cytoskeleton formation and resistance to mechanical stress, and plays a role in the regulation of diseases such as squamous cell lung carcinoma and breast cancer (50, 51). Zhang et al. found that knockout of KRT81 led to downregulation of CXCL8, suggesting that KRT81 may affect CCM formation by participating in CXCL8-related signaling pathways (52). It may also influence NK cell migration and immune deposition by affecting NK cell structure. CXCL14, a chemokine, primarily regulates the recruitment and activation of immune cells (e.g., dendritic cells, NK cells) and is involved in angiogenesis and inflammatory microenvironment regulation (53). Wang et al. found that CXCL14 promotes NK cell migration and infiltration in head and neck squamous cell carcinoma (54). We observed that CXCL14 was downregulated in both NK cells and neutrophils, which may affect their correct migration and lead to improper accumulation in CCM lesion tissues. Neutrophils exhibited upregulation of EPSTI1 and downregulation of CXCL14. EPSTI1 (Epithelial Stromal Interaction 1) was initially discovered in breast cancer and is involved in epithelial-mesenchymal transition (EMT), inflammation, and immune regulation (55). Bei et al. found that EPSTI1 promotes monocyte and endothelial cell adhesion by upregulating VCAM-1 and ICAM-1 expression (56). Although we found its upregulation in neutrophils, it may share the same regulatory pathways with monocytes, which is consistent with our findings from cell communication analysis, where VCAM and ICAM pathways were upregulated. ADH1B (Alcohol Dehydrogenase 1B) belongs to the alcohol dehydrogenase family and is involved in ethanol metabolism (57). Jiang et al. found that overexpression of ADH1B can deactivate the MAPK signaling pathway (58). This suggests that downregulation of ADH1B may activate the MAPK pathway, contributing to the formation of CCM lesion tissues. The abnormal expression of monocytes, NK cells, and neutrophils is closely related to the dysregulation of their respective gene expression. Further investigation into how these genes affect the corresponding cell subpopulations will help us better understand the mechanisms underlying CCM formation.

Overall, our study provides the single-cell resolution map of the CCM cellular microenvironment, uncovering key immune cell alterations, dysregulated signaling pathways, and intercellular communication changes. These findings contribute to a deeper understanding of CCM pathogenesis and highlight potential therapeutic targets. Future research should validate these findings using larger patient cohorts and functional experiments to explore targeted therapeutic interventions for CCM.

### Limitations of the study

There are still some limitations in this study. The difficulty of obtaining CCM tissue through surgery meant we only acquired four samples, which limited the statistical power of our analysis of cell subpopulations. Future studies should expand the cohort to validate the patterns of change in key cell types. Many of the genes and pathways identified in this study have not been previously researched in the context of CCM, and future experimental validation of these specific genes and pathways is needed.

## Data Availability

All single-cell RNA sequencing data generated in this study have been deposited in the NCBI Gene Expression Omnibus (GEO) database under the accession number GSE294555.

## References

[1] Cavalcanti DD Kalani MY Martirosyan NL Eales J Spetzler RF Preul MC. Cerebral cavernous malformations: from genes to proteins to disease. J Neurosurg. (2012) 116:122–32. doi: 10.3171/2011.8.Jns101241 21962164

[2] Zabramski JM Wascher TM Spetzler RF Johnson B Golfinos J Drayer BP. The natural history of familial cavernous malformations: results of an ongoing study. J Neurosurg. (1994) 80:422–32. doi: 10.3171/jns.1994.80.3.0422 8113854

[3] Clatterbuck RE Eberhart CG Crain BJ Rigamonti D. Ultrastructural and immunocytochemical evidence that an incompetent blood-brain barrier is related to the pathophysiology of cavernous malformations. J Neurol Neurosurg Psychiatry. (2001) 71:188–92. doi: 10.1136/jnnp.71.2.188 PMC1737494 11459890

[4] Batra S Lin D Recinos PF Zhang J Rigamonti D. Cavernous malformations: natural history, diagnosis and treatment. Nat Rev Neurol. (2009) 5:659–70. doi: 10.1038/nrneurol.2009.177 19953116

[5] Croft J Grajeda B Gao L Abou-Fadel J Badr A Sheng V. Whole-genome omics elucidates the role of CCM1 and progesterone in cerebral cavernous malformations within cmPn networks. Diagn (Basel). (2024) 14(17):1895. doi: 10.3390/diagnostics14171895 PMC11394482 39272679

[6] Johnson EW Iyer LM Rich SS Orr HT Gil-Nagel A Kurth JH. Refined localization of the cerebral cavernous malformation gene (CCM1) to a 4-cM interval of chromosome 7q contained in a well-defined YAC contig. Genome Res. (1995) 5:368–80. doi: 10.1101/gr.5.4.368 8750196

[7] Craig HD Günel M Cepeda O Johnson EW Ptacek L Steinberg GK. Multilocus linkage identifies two new loci for a mendelian form of stroke, cerebral cavernous malformation, at 7p15–13 and 3q25.2-27. Hum Mol Genet. (1998) 7:1851–8. doi: 10.1093/hmg/7.12.1851 9811928

[8] Hilder TL Malone MH Bencharit S Colicelli J Haystead TA Johnson GL. Proteomic identification of the cerebral cavernous malformation signaling complex. J Proteome Res. (2007) 6:4343–55. doi: 10.1021/pr0704276 17900104

[9] Hong T Xiao X Ren J Cui B Zong Y Zou J. Somatic MAP3K3 and PIK3CA mutations in sporadic cerebral and spinal cord cavernous malformations. Brain. (2021) 144:2648–58. doi: 10.1093/brain/awab117 33729480

[10] Ma X Zhao H Shan J Long F Chen Y Chen Y. PDCD10 interacts with Ste20-related kinase MST4 to promote cell growth and transformation via modulation of the ERK pathway. Mol Biol Cell. (2007) 18:1965–78. doi: 10.1091/mbc.e06-07-0608 PMC1877091 17360971

[11] Li A Dubey S Varney ML Dave BJ Singh RK. IL-8 directly enhanced endothelial cell survival, proliferation, and matrix metalloproteinases production and regulated angiogenesis. J Immunol. (2003) 170:3369–76. doi: 10.4049/jimmunol.170.6.3369 12626597

[12] Yau ACY Globisch MA Onyeogaziri FC Conze LL Smith R Jauhiainen S. Inflammation and neutrophil extracellular traps in cerebral cavernous malformation. Cell Mol Life Sci. (2022) 79:206. doi: 10.1007/s00018-022-04224-2 35333979 PMC8949649

[13] Abou-Fadel J Vasquez M Grajeda B Ellis C Zhang J. Systems-wide analysis unravels the new roles of CCM signal complex (CSC). Heliyon. (2019) 5(12):e02899. doi: 10.1016/j.heliyon.2019.e02899 31872111 PMC6909108

[14] Koskimäki J Zhang D Li Y Saadat L Moore T Lightle R. Transcriptome clarifies mechanisms of lesion genesis versus progression in models of Ccm3 cerebral cavernous malformations. Acta Neuropathol Commun. (2019) 7:132. doi: 10.1186/s40478-019-0789-0 31426861 PMC6699077

[15] Tang F Barbacioru C Wang Y Nordman E Lee C Xu N. mRNA-Seq whole-transcriptome analysis of a single cell. Nat Methods. (2009) 6:377–82. doi: 10.1038/nmeth.1315 19349980

[16] Mondéjar R Solano F Rubio R Delgado M Pérez-Sempere A González-Meneses A. Mutation prevalence of cerebral cavernous malformation genes in Spanish patients. PloS One. (2014) 9:e86286. doi: 10.1371/journal.pone.0086286 24466005 PMC3900513

[17] Ziegenhain C Vieth B Parekh S Reinius B Guillaumet-Adkins A Smets M. Comparative analysis of single-cell RNA sequencing methods. Mol Cell. (2017) 65:631–643.e4. doi: 10.1016/j.molcel.2017.01.023 28212749

[18] Cole JE Park I Ahern DJ Kassiteridi C Danso Abeam D Goddard ME. Immune cell census in murine atherosclerosis: cytometry by time of flight illuminates vascular myeloid cell diversity. Cardiovasc Res. (2018) 114:1360–71. doi: 10.1093/cvr/cvy109 PMC6054192 29726984

[19] Zhang H Li B Huang Q López-Giráldez F Tanaka Y Lin Q. Mitochondrial dysfunction induces ALK5-SMAD2-mediated hypovascularization and arteriovenous malformations in mouse retinas. Nat Commun. (2022) 13:7637. doi: 10.1038/s41467-022-35262-w 36496409 PMC9741628

[20] Abou-Fadel J Smith M Falahati K Zhang J. Comparative omics of CCM signaling complex (CSC). Chin Neurosurg J. (2020) 6:4. doi: 10.1186/s41016-019-0183-6 32922933 PMC7398211

[21] Koskimäki J Girard R Li Y Saadat L Zeineddine HA Lightle R. Comprehensive transcriptome analysis of cerebral cavernous malformation across multiple species and genotypes. JCI Insight. (2019) 4(3):e126167. doi: 10.1172/jci.insight.126167 30728328 PMC6413775

[22] Harrison PW Amode MR Austine-Orimoloye O Azov AG Barba M Barnes I. Ensembl 2024. Nucleic Acids Res. (2024) 52:D891–d899. doi: 10.1093/nar/gkad1049 37953337 PMC10767893

[23] Zheng GX Terry JM Belgrader P Ryvkin P Bent ZW Wilson R. Massively parallel digital transcriptional profiling of single cells. Nat Commun. (2017) 8:14049. doi: 10.1038/ncomms14049 28091601 PMC5241818

[24] Hao Y Hao S Andersen-Nissen E Mauck WM 3rd Zheng S Butler A. Integrated analysis of multimodal single-cell data. Cell. (2021) 184:3573–3587.e29. doi: 10.1016/j.cell.2021.04.048 34062119 PMC8238499

[25] Aran D Looney AP Liu L Wu E Fong V Hsu A. Reference-based analysis of lung single-cell sequencing reveals a transitional profibrotic macrophage. Nat Immunol. (2019) 20:163–72. doi: 10.1038/s41590-018-0276-y PMC6340744 30643263

[26] Yu G Wang LG Han Y He QY. clusterProfiler: an R package for comparing biological themes among gene clusters. Omics. (2012) 16:284–7. doi: 10.1089/omi.2011.0118 PMC3339379 22455463

[27] Kanehisa M Goto S. KEGG: kyoto encyclopedia of genes and genomes. Nucleic Acids Res. (2000) 28:27–30. doi: 10.1093/nar/28.1.27 10592173 PMC102409

[28] Ashburner M Ball CA Blake JA Botstein D Butler H Michael Cherry J. Gene ontology: tool for the unification of biology. The Gene Ontology Consortium. Nat Genet. (2000) 25:25–9. doi: 10.1038/75556 PMC3037419 10802651

[29] Jin S Plikus MV Nie Q. CellChat for systematic analysis of cell-cell communication from single-cell transcriptomics. Nat Protoc. (2025) 20:180–219. doi: 10.1038/s41596-024-01045-4 39289562

[30] Matsushima K Baldwin ET Mukaida N. Interleukin-8 and MCAF: novel leukocyte recruitment and activating cytokines. Chem Immunol. (1992) 51:236–65. 1567543

[31] Brat DJ Bellail AC Van Meir EG. The role of interleukin-8 and its receptors in gliomagenesis and tumoral angiogenesis. Neuro Oncol. (2005) 7:122–33. doi: 10.1215/s1152851704001061 PMC1871893 15831231

[32] Liu Q Li A Tian Y Wu JD Liu Y Li T. The CXCL8-CXCR1/2 pathways in cancer. Cytokine Growth Factor Rev. (2016) 31:61–71. doi: 10.1016/j.cytogfr.2016.08.002 27578214 PMC6142815

[33] Huo R Yang Y Sun Y Zhou Q Zhao S Mo Z. Endothelial hyperactivation of mutant MAP3K3 induces cerebral cavernous malformation enhanced by PIK3CA GOF mutation. Angiogenesis. (2023) 26:295–312. doi: 10.1007/s10456-023-09866-9 36719480

[34] Knall C Young S Nick JA Buhl AM Worthen GS Johnson GL. Interleukin-8 regulation of the Ras/Raf/mitogen-activated protein kinase pathway in human neutrophils. J Biol Chem. (1996) 271:2832–8. doi: 10.1074/jbc.271.5.2832 8576262

[35] Whitehead KJ Chan AC Navankasattusas S. The cerebral cavernous malformation signaling pathway promotes vascular integrity via Rho GTPases. Nat Med. (2009) 15:177–84. doi: 10.1038/nm.1911 PMC2767168 19151728

[36] Schraufstatter IU Chung J Burger M. IL-8 activates endothelial cell CXCR1 and CXCR2 through Rho and Rac signaling pathways. Am J Physiol Lung Cell Mol Physiol. (2001) 280:L1094–103. doi: 10.1152/ajplung.2001.280.6.L1094 11350788

[37] Ayata C Kim H Morrison L Liao JK Gutierrez J Lopez-Toledano M. Role of rho-associated kinase in the pathophysiology of cerebral cavernous malformations. Neurol Genet. (2024) 10:e200121. doi: 10.1212/nxg.0000000000200121 38179414 PMC10766084

[38] Li Y Srinath A Alcazar-Felix RJ Hage S Bindal A Lightle R. Inflammatory mechanisms in a neurovascular disease: cerebral cavernous malformation. Brain Sci. (2023) 13(9):1336. doi: 10.3390/brainsci13091336 37759937 PMC10526329

[39] Scimone C Alibrandi S Donato L Alafaci C Germanò A Vinci SL. Editome landscape of CCM-derived endothelial cells. RNA Biol. (2022) 19:852–65. doi: 10.1080/15476286.2022.2091306 PMC9248949 35771000

[40] Zhang J Basu S Rigamonti D Dietz HC Clatterbuck RE. Krit1 modulates beta 1-integrin-mediated endothelial cell proliferation. Neurosurgery. (2008) 63:571–8. doi: 10.1227/01.Neu.0000325255.30268.B0 18812969

[41] Liu H Rigamonti D Badr A Zhang J. Ccm1 assures microvascular integrity during angiogenesis. Transl Stroke Res. (2010) 1:146–53. doi: 10.1007/s12975-010-0010-z PMC3090208 21562623

[42] Liu H Rigamonti D Badr A Zhang J. Ccm1 regulates microvascular morphogenesis during angiogenesis. J Vasc Res. (2011) 48:130–40. doi: 10.1159/000316851 PMC3219476 20926893

[43] Renz M Otten C Faurobert E. Regulation of β1 integrin-Klf2-mediated angiogenesis by CCM proteins. Dev Cell. (2015) 32:181–90. doi: 10.1016/j.devcel.2014.12.016 25625207

[44] Malinverno M Maderna C Abu Taha A Corada M Orsenigo F Valentino M. Endothelial cell clonal expansion in the development of cerebral cavernous malformations. Nat Commun. (2019) 10:2761. doi: 10.1038/s41467-019-10707-x 31235698 PMC6591323

[45] Detter MR Snellings DA Marchuk DA. Cerebral cavernous malformations develop through clonal expansion of mutant endothelial cells. Circ Res. (2018) 123:1143–51. doi: 10.1161/circresaha.118.313970 PMC6205520 30359189

[46] Ren AA Snellings DA Su YS Hong CC Castro M Tang AT. PIK3CA and CCM mutations fuel cavernomas through a cancer-like mechanism. Nature. (2021) 594:271–6. doi: 10.1038/s41586-021-03562-8 PMC8626098 33910229

[47] Pilz RA Skowronek D Mellinger L Bekeschus S Felbor U Rath M. Endothelial differentiation of CCM1 knockout iPSCs triggers the establishment of a specific gene expression signature. Int J Mol Sci. (2023) 24(4):3993. doi: 10.3390/ijms24043993 36835400 PMC9963194

[48] Johansen FE Braathen R Brandtzaeg P. Role of J chain in secretory immunoglobulin formation. Scand J Immunol. (2000) 52:240–8. doi: 10.1046/j.1365-3083.2000.00790.x 10972899

[49] Shukunami C Oshima Y Hiraki Y. Chondromodulin-I and tenomodulin: a new class of tissue-specific angiogenesis inhibitors found in hypovascular connective tissues. Biochem Biophys Res Commun. (2005) 333:299–307. doi: 10.1016/j.bbrc.2005.05.133 15950187

[50] Campayo M Navarro A Viñolas N. A dual role for KRT81: a miR-SNP associated with recurrence in non-small-cell lung cancer and a novel marker of squamous cell lung carcinoma. PloS One. (2011) 6:e22509. doi: 10.1371/journal.pone.0022509 21799879 PMC3143163

[51] Yan Z Zhong Z Shi C Feng M Feng X Liu T. The prognostic marker KRT81 is involved in suppressing CD8 + T cells and predicts immunotherapy response for triple-negative breast cancer. Cancer Biol Ther. (2024) 25:2355705. doi: 10.1080/15384047.2024.2355705 38778753 PMC11123506

[52] Zhang K Liang Y Zhang W Zeng N Tang S Tian R. KRT81 knockdown inhibits Malignant progression of melanoma through regulating interleukin-8. DNA Cell Biol. (2021) 40:1290–7. doi: 10.1089/dna.2021.0317 34591651

[53] Tsuji K Tanegashima K Sato K Sakamoto K Shigenaga A Inokuma T. Efficient one-pot synthesis of CXCL14 and its derivative using an N-sulfanylethylanilide peptide as a peptide thioester equivalent and their biological evaluation. Bioorg Med Chem. (2015) 23:5909–14. doi: 10.1016/j.bmc.2015.06.064 26187016

[54] Wang H Nan S Wang Y Xu C. CDX2 enhances natural killer cell-mediated immunotherapy against head and neck squamous cell carcinoma through up-regulating CXCL14. J Cell Mol Med. (2021) 25:4596–607. doi: 10.1111/jcmm.16253 PMC8107099 33733587

[55] Nielsen HL Rønnov-Jessen L Villadsen R Petersen OW. Identification of EPSTI1, a novel gene induced by epithelial-stromal interaction in human breast cancer. Genomics. (2002) 79:703–10. doi: 10.1006/geno.2002.6755 11991720

[56] Bei YR Zhang SC Song Y Tang ML Zhang KL Jiang M. EPSTI1 promotes monocyte adhesion to endothelial cells *in vitro* via upregulating VCAM-1 and ICAM-1 expression. Acta Pharmacol Sin. (2023) 44:71–80. doi: 10.1038/s41401-022-00923-5 35778487 PMC9812988

[57] Edenberg HJ. The genetics of alcohol metabolism: role of alcohol dehydrogenase and aldehyde dehydrogenase variants. Alcohol Res Health. (2007) 30:5–13. 17718394 PMC3860432

[58] Jiang C Liu R Wu X. Alcohol dehydrogenase-1B represses the proliferation, invasion and migration of breast cancer cells by inactivating the mitogen-activated protein kinase signalling pathway. J Physiol Pharmacol. (2023) 74(5):10.26402/jpp.2023.5.10. doi: 10.26402/jpp.2023.5.10 38085522

