# Supplementary material for: Single-cell transcriptome profiling reveals dynamic cell populations and immune infiltration in cerebral cavernous malformation
Source: Front Immunol. 2025 May 30;16:1592343. doi: 10.3389/fimmu.2025.1592343 (PMC12163010; doi:10.3389/fimmu.2025.1592343)
Supplement: Supplementary file 1 [file DataSheet1.pdf]

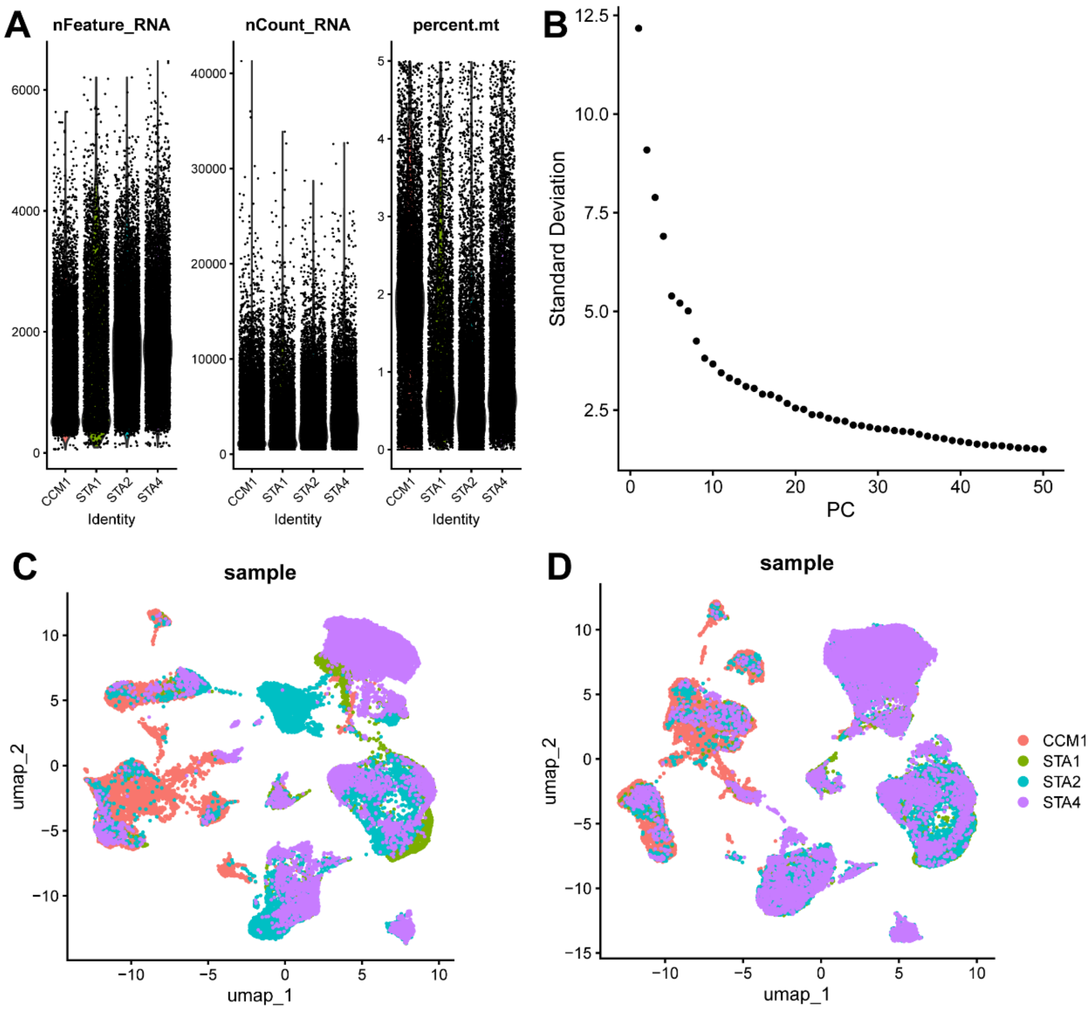

Figure S1. Single-cell data processing results. A, Data quality control metrics. Distribution of (left) RNA feature count, (middle) RNA count, and (right) percentage of mitochondrial genes across different sample identities: CCM1, STA1, STA2, and STA4. **B,** Scree plot of the standard deviation of each principal component. the variance in the principal components plateaued when the number of components reached 20. **C,** UMAP clustering of cells by sample identity (before batch effect correction). **D,** UMAP clustering of cells by sample identity (after batch effect correction).

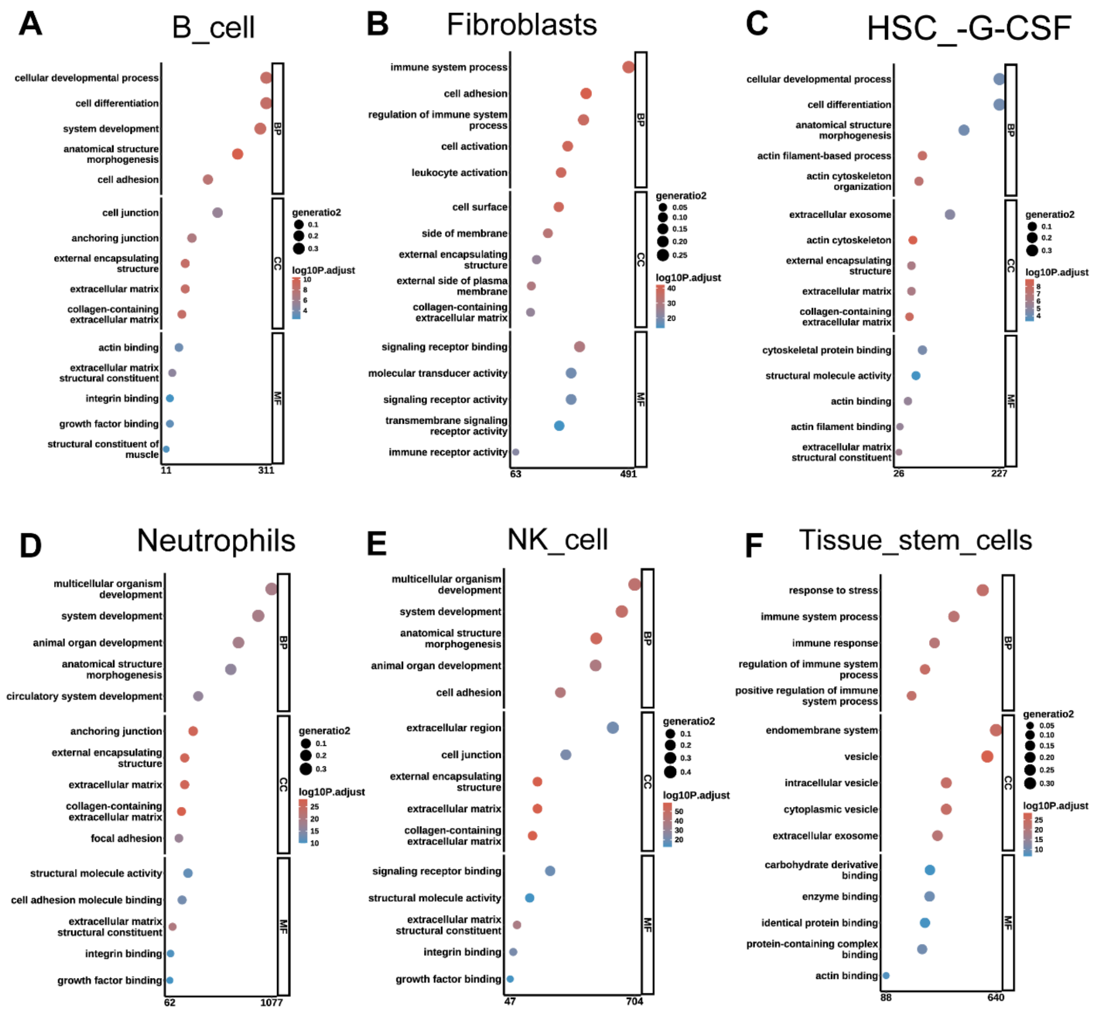

Figure S2. GO enrichment results of B cells, Fibroblasts, HSC-G-CSF, Neutrophils, NK cells and Tissue stem cells. A, B cells had 279 significantly enriched GO terms, including 214 BP terms, 50 CC terms and 15 MF terms. **B,** Fibroblasts had 1,666 significantly enriched GO terms, including 1,396 BP terms, 143 CC terms and 127 MF terms. **C,** HSC-G-CSF had 174 significantly enriched GO terms, including 94 BP terms, 65 CC terms and 15 MF terms. **D,** Neutrophils had 984 significantly enriched GO terms, including 751 BP terms, 160 CC terms and 73 MF terms. **E,** NK cells had 1,663 significantly enriched GO terms, including 1,426 BP terms, 154 CC terms and 83 MF terms. **F,** Tissue stem cells had 1,550 significantly enriched GO terms, including 1,285 BP terms, 167 CC terms and 98 MF terms.

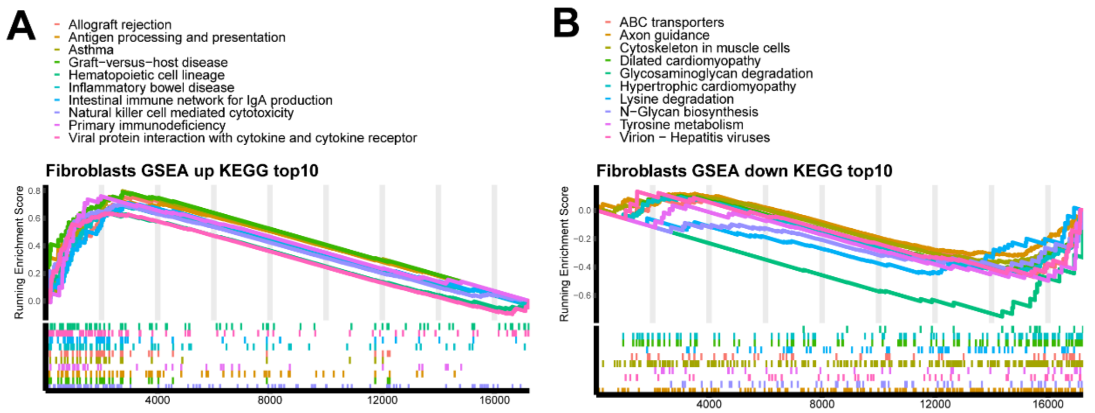

Figure S3. KEGG enrichment results of Fibroblasts. A, Top 10 upregulated pathways in Fibroblasts. The most significant upregulated signaling pathway is Natural Killer cell-mediated cytotoxicity. **B,** Top 10 downregulated pathways in Fibroblasts. The most significant downregulated signaling pathway is Axon guidance.

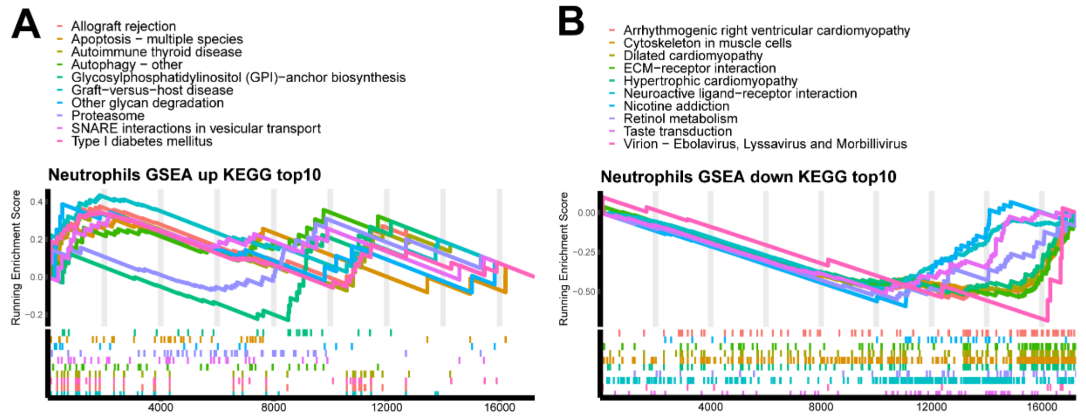

Figure S4. KEGG enrichment results of Neutrophils. A, Top 10 upregulated pathways in Neutrophils. The most significant upregulated signaling pathway is Graft-versus-host disease. **B,** Top 10 downregulated pathways in Neutrophils. The most significant downregulated signaling pathway is Taste transduction.

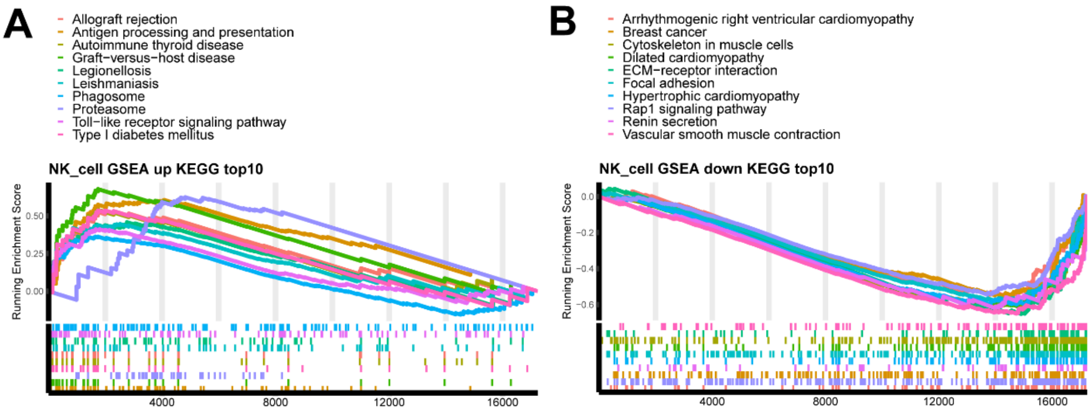

Figure S5. KEGG enrichment results of NK cells. A, Top 10 upregulated pathways

in NK cells. The most significant upregulated signaling pathway is Antigen processing and presentation. **B,** Top 10 downregulated pathways in NK cells. The most significant downregulated signaling pathway is Arrhythmogenic right ventricular cardiomyopathy.

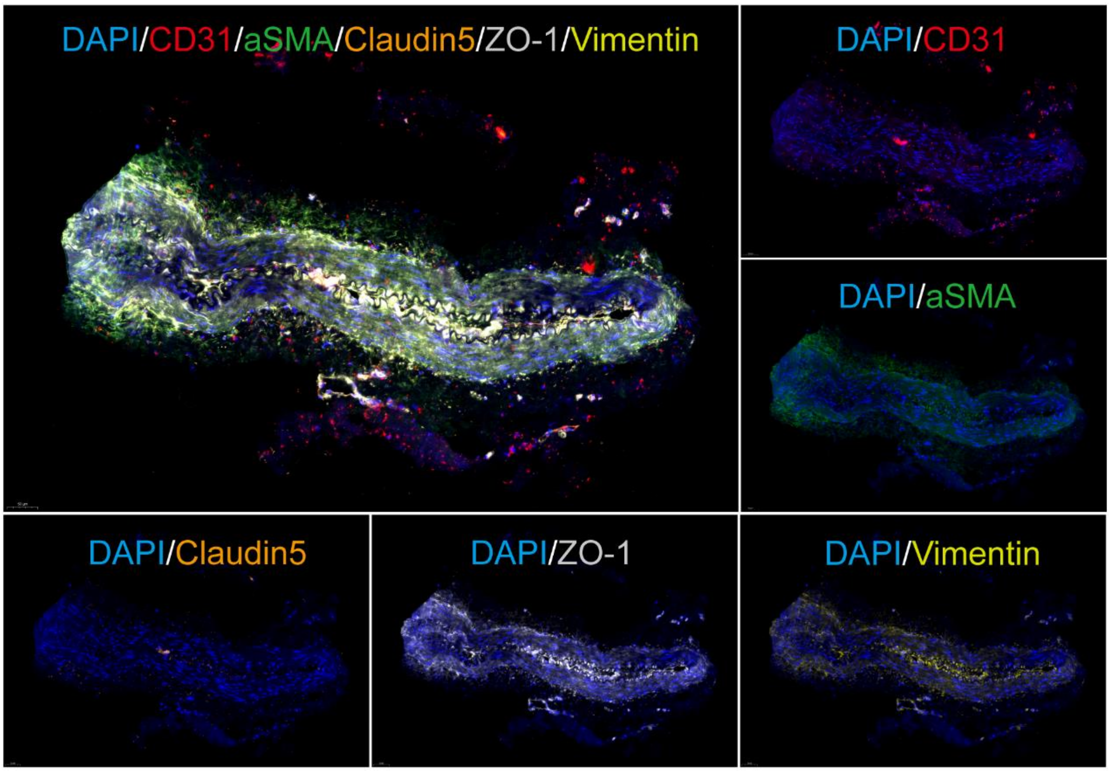

Figure S6. Multiplex immunofluorescence staining for control. The tissue was stained by different antibodies. CD31, an antibody to mark endothelial cells, was represented as red fluorescence. For smooth muscle cell, we used antibody aSMA, which was shown in green color. We used Claudin5 and Zo-1700 to mark blood-brain barrier or tight junction, which was stained in orange and white color respectively. Vimentin was used to mark fibroblasts and was stained in yellow. Scale bar = 50 um.
